# Genetic Diversity of Salt Tolerance in *Miscanthus*

**DOI:** 10.3389/fpls.2017.00187

**Published:** 2017-02-14

**Authors:** Chang-Lin Chen, Hanneke van der Schoot, Shiva Dehghan, Claire L. Alvim Kamei, Kai-Uwe Schwarz, Heike Meyer, Richard G. F. Visser, C. Gerard van der Linden

**Affiliations:** ^1^Plant Breeding, Wageningen University and ResearchWageningen, Netherlands; ^2^Graduate School Experimental Plant Science, Wageningen University and ResearchWageningen, Netherlands; ^3^Department of Comparative Development and Genetics, Max Planck Institute for Plant Breeding ResearchCologne, Germany; ^4^Julius Kühn-Institute, Institute for Crop and Soil ScienceBraunschweig, Germany

**Keywords:** *Miscanthus*, salt tolerance, osmotic stress, ionic stress, ion homeostasis

## Abstract

*Miscanthus* is a woody rhizomatous C4 grass that can be used as a CO_2_ neutral biofuel resource. It has potential to grow in marginal areas such as saline soils, avoiding competition for arable lands with food crops. This study explored genetic diversity for salt tolerance in *Miscanthus* and discovered mechanisms and traits that can be used to improve the yield under salt stress. Seventy genotypes of *Miscanthus* (including 57 *M. sinensis*, 5 *M. sacchariflorus*, and 8 hybrids) were evaluated for salt tolerance under saline (150 mM NaCl) and normal growing conditions using a hydroponic system. Analyses of shoot growth traits and ion concentrations revealed the existence of large variation for salt tolerance in the genotypes. We identified genotypes with potential for high biomass production both under control and saline conditions that may be utilized for growth under marginal, saline conditions. Several relatively salt tolerant genotypes had clearly lower Na^+^ concentrations and showed relatively high K^+^/Na^+^ ratios in the shoots under salt stress, indicating that a Na^+^ exclusion mechanism was utilized to prevent Na^+^ accumulation in the leaves. Other genotypes showed limited reduction in leaf expansion and growth rate under saline conditions, which may be indicative of osmotic stress tolerance. The genotypes demonstrating potentially different salt tolerance mechanisms can serve as starting material for breeding programs aimed at improving salinity tolerance of *Miscanthus*.

## Introduction

*Miscanthus* is a C4 perennial grass originating from Southeast Asia, the Pacific islands, and tropical Africa. The genus *Miscanthus* has a basic chromosome number of 19, and includes the nominally diploid species *Miscanthus sinensis* (2N = 2x = 38) and tetraploid species *Miscanthus sacchariflorus* (2N = 4x = 76) plus a triploid interspecific hybrid, *Miscanthus* × *giganteus* (3n = 3x = 57). This hybrid was identified as a good candidate for energy production by direct combustion (Zub and Brancourt-Hulmel, 2010). However, *Miscanthus* × *giganteus* has several disadvantages. Since *Miscanthus* × *giganteus* is a sterile triploid, it is difficult to improve its genetics by crossing. In addition, its sterility requires propagation from rhizomes or tissue culture, which is relatively more expensive than from seeds (Greef and Deuter, 1993). To screen and explore natural genetic diversity from other sources is therefore important for genetic improvement of the crop. A good alternative for breeding purposes is the diploid species *M. sinensis*. An important breeding goal for any bioenergy crop and also *Miscanthus* is to achieve economically viable yields in marginal lands, thus avoiding competition with food crops and interfering with food security (Somerville et al., 2010).

High soil salinity is one of the major constraints of crop growth because it decreases crop yield and quality. Almost 20% of the world's irrigated land is adversely influenced by salinity (Flowers and Yeo, 1995; Munns and Tester, 2008; Rengasamy, 2010b; Qadir et al., 2014), and the problem of soil salinity is further increasing because of poor drainage and climatic change (Bennett and Khush, 2003). Salinity affects plant growth because of osmotic stress, ionic stress, and nutritional imbalance (Ashraf and Harris, 2004; Munns and Tester, 2008). Osmotic stress affects growth immediately and is in saline soils caused by limitation of water uptake resulting from the high salt concentration in the soil. Ionic stress develops over time and is due to ion accumulation within the shoots. Osmotic stress accounts for roughly 75% of the biomass decrease under salt stress, and ionic stress reduces it by another 20% (Munns and Tester, 2008). The strong effect of salinity on crop yield makes salinity tolerance in crops an important target for breeding. However, breeding for salt tolerance is not straightforward due to its genetic complexity.

Salt stress affects all the major processes underlying plant growth, including lipid and energy metabolism, photosynthesis, and protein synthesis (Parida and Das, 2005). This leads to reduction in transpiration, chlorophyll content, tiller number, and biomass (Hassanein, 1999; Chartzoulakis and Klapaki, 2000). The altered water status and unbalanced ion homeostasis resulting from saline conditions induce several mechanisms to reduce damage in the plant. Osmotic tolerance can be achieved by adapting water uptake properties of the roots, plant hydraulics, and by adjusting the plant's osmotic potential. Production of compatible solutes like proline (Khatkar and Kuhad, 2000), glycine betaine (Khan et al., 2000; Wang and Nii, 2000), sugars (Kerepesi and Galiba, 2000), and polyols (Bohnert et al., 1995; Zhifang and Loescher, 2003) facilitates osmotic adjustment or osmotic protection. To avoid toxic ion concentrations in shoots, plants exclude access sodium and chloride ions from the shoot. Bread wheat for instance has a low rate of Na^+^ transport to the shoot and maintains a high ratio of K^+^/Na^+^ in the leaves, which contributes to salt tolerance, while surum wheat is more salt-sensitive due to its poor ability to exclude Na^+^ from the shoot (Gorham et al., 1990). Shoot exclusion was shown to be facilitated by a members of the high-affinity K^+^ transporter (HKT) family (HKT1;5) that can take Na^+^ from the xylem into the parenchyma cells to minimize the accumulation of Na^+^ in the shoot (Conde et al., 2011). Tissue tolerance to high salt concentrations is a mechanis often utilized by halophytes, and it can be achieved by compartmentalization of Na^+^ and Cl^−^ in cellular organelles like the vacuoles (Adams et al., 1992) and involves tonoplast Na^+^/H^+^ antiporters (NHX) that regulate cytosolic Na^+^ concentration and pH (Bassil et al., 2012). In mature leaves, senescence may reflect the toxic effect of high levels of Na^+^ concentration and low tissue tolerance to Na^+^ (Munns and James, 2003). The combination of accumulation of Na^+^ in leaves, lack of necrosis, and relatively little reduction of biomass can be indicative of tissue tolerance (Munns and James, 2003; Rajendran et al., 2009).

Salt stress not only affects the quantity but also the quality of *Miscanthus* biomass. *Miscanthus* genotypes with less ions in the harvestable biomass are particularly important because high concentrations of minerals can be corrosive to combustion equipment (Jorgensen, 1997). Thus, it is essential for *Miscanthus* to produce stable biomass with low ion concentrations under salt stress. Only few studies have been done in relation to salt tolerance of *Miscanthus* (Li et al., 2014; Plazek et al., 2014; Sun et al., 2014; Stavridou et al., 2016), and *Miscanthus* may be considered a moderately salt tolerant crop with salt concentrations higher than 100 mM NaCl (approximately 10 dS/m) reducing crop yields considerably. Until now the genetic diversity of salt tolerance in *Miscanthus* germplasm has not been investigated, although Sun et al. (2014) indicate that *M. sinensis* may harbor significant genetic variation for salt tolerance. The current study aims to explore genetic diversity of *Miscanthus* breeding material to identify genotypes for cultivation in saline soils, and genotypes that harbor salt tolerance traits and can serve as material for improvement of *Miscanthus* salt tolerance. The results showed that several genotypes with relatively high salt tolerance appeared to rely on different mechanisms, offering opportunities for breeding programs aimed at improved tolerance of *Miscanthus*.

## Materials and methods

### Plant materials

Seventy genotypes of *Miscanthus* were evaluated for salt tolerance (Table 1). The set included 57 *M. sinensis*, 5 *M. sacchariflorus* and eight hybrids (OPM-9 is *Miscanthus* × *giganteus*) and each genotype was cloned and propagated by tissue culture. The genotypes were supplied by different sources (Aberystwyth University, Institute for Agricultural and Fisheries Research ILVO, and Wageningen University & Research). Two genotypes were tested in a pilot experiment to establish optimal experimental conditions.

**Table 1:** ***Miscanthus* genotypes screened for salt tolerance**.

**No**.	**Supplier**	**Genotype**
OPM-4	IBERS	*M. sacchariflorus*
OPM-5	IBERS	Hybrid (*M. sinensis* × *M. sacchariflorus*)
OPM-6	IBERS	Hybrid (*M. sacchariflorus* × *M. sinensis*)
OPM-7	IBERS	Hybrid (*M. sacchariflorus* × *M. sinensis*)
OPM-8	IBERS	Hybrid (*M. sacchariflorus* × *M. sinensis*)
OPM-9	IBERS	Hybrid (*Miscanthus* × *giganteus*)
OPM-10	IBERS	Hybrid (*M. sacchariflorus* × *M. sinensis*)
OPM-11	IBERS	*M. sinensis*
OPM-13^*^	WUR	*M. sinensis*
OPM-16	IBERS	Hybrid (*M. sacchariflorus* × *M. sinensis*)
OPM-19	IBERS	*M. sacchariflorus*
OPM-20	IBERS	Hybrid (*M. sacchariflorus* × *M. sinensis*)
OPM-24	IBERS	*M. sacchariflorus*
OPM-26	IBERS	*M. sacchariflorus*
OPM-30	IBERS	*M. sinensis*
OPM-31	IBERS	*M. sinensis*
OPM-32	IBERS	*M. sinensis*
OPM-33	IBERS	*M. sinensis*
OPM-34	IBERS	*M. sacchariflorus*
OPM-37	WUR	*M. sinensis*
OPM-38^*^	WUR	*M. sinensis*
OPM-41	WUR	*M. sinensis*
OPM-42	WUR	*M. sinensis*
OPM-44	WUR	*M. sinensis*
OPM-45	WUR	*M. sinensis*
OPM-47	WUR	*M. sinensis*
OPM-48	WUR	*M. sinensis*
OPM-49	WUR	*M. sinensis*
OPM-50	WUR	*M. sinensis*
OPM-56	WUR	*M. sinensis*
OPM-57	WUR	*M. sinensis*
OPM-58	WUR	*M. sinensis*
OPM-59	WUR	*M. sinensis*
OPM-62	WUR	*M. sinensis*
OPM-64	WUR	*M. sinensis*
OPM-65	WUR	*M. sinensis*
OPM-66	WUR	*M. sinensis*
OPM-67	WUR	*M. sinensis*
OPM-68	WUR	*M. sinensis*
OPM-69	WUR	*M. sinensis*
OPM-71	WUR	*M. sinensis*
OPM-72	WUR	*M. sinensis*
OPM-73	WUR	*M. sinensis*
OPM-74	WUR	*M. sinensis*
OPM-75	WUR	*M. sinensis*
OPM-76	WUR	*M. sinensis*
OPM-77	WUR	*M. sinensis*
OPM-78	WUR	*M. sinensis*
OPM-79	WUR	*M. sinensis*
OPM-81	IBERS	*M. sinensis*
OPM-82	WUR	*M. sinensis*
OPM-83	WUR	*M. sinensis*
OPM-84	WUR	*M. sinensis*
OPM-86	WUR	*M. sinensis*
OPM-87	WUR	*M. sinensis*
OPM-88	WUR	*M. sinensis*
OPM-89	WUR	*M. sinensis*
OPM-90	WUR	*M. sinensis*
OPM-91	WUR	*M. sinensis*
OPM-92	WUR	*M. sinensis*
OPM-94	WUR	*M. sinensis*
OPM-96	IBERS	*M. sinensis*
OPM-97	IBERS	*M. sinensis*
OPM-98	WUR	*M. sinensis*
OPM-99	WUR	*M. sinensis*
OPM-100	ILVO	*M. sinensis*
OPM-101	WUR	*M. sinensis*
OPM-103	WUR	*M. sinensis*
OPM-104	WUR	*M. sinensis*
OPM-107	WUR	*M. sinensis*
OPM-108	WUR	*M. sinensis*
OPM-109	IBERS	*M. sinensis*

*The OPM code for the genotypes was used within the EU project OPTIMISC*.

*IBERS, Institute of Biological, Environmental, and Rural Sciences; Aberystwyth University, UK; ILVO, The Institute for Agricultural and Fisheries Research, Belgium; WUR, Wageningen University & Research; The Netherlands*.

* *in pilot experiment*.

### Pilot experiment

Two genotypes (OPM-13 and OPM-38) were grown under different levels of salinity (0 mM, 125 mM, and 250 mM NaCl). The seedlings were propagated *in vitro*, transferred to the hydroponics system and allowed to acclimate for 1 week. The hydroponics system consisted of containers (22 L, 40 cm length, 30 cm width and 20 cm height) that can hold up to 12 *Miscanthus* plants. A maximum of 16 containers can be connected as a unit to a single reservoir, with capacity of 500l nutrient solution. For the pilot experiment, three units were used for the three different salt levels, each with two connected containers. The nutrient solution was half-strength modified Hoagland's solution (Supplemental Table 1), maintained at pH 5.8 and refreshed weekly. Seedlings with four leaves were selected and transferred to the hydroponics containers. Each container had two genotypes in four replications (8 plants). After 1 week of acclimation, NaCl was added to the nutrient solutions of two of the units with a 25 mM daily increment until a concentration of 125 mM NaCl. Only one of those units received two more additions of 62.5 mM NaCl to reach 250 mM NaCl. The average day/night temperatures were set at 25/18°C, and the photoperiod regime was 16 h light and 8 h dark. Greenhouse environmental humidity was controlled at 70%. Additional lighting (100 Wm^−2^) was used when the incoming shortwave radiation was below 200 Wm^−2^. After 2 weeks of salt treatment the shoot dry weight and Na^+^ and Cl^−^ concentrations of the shoots were measured and evaluated.

### Main experiment design

Seedlings from the 70 genotypes were propagated *in vitro* for 6 weeks, and allowed to form roots. Then they were transferred to the greenhouse and allowed to acclimate for 2 weeks on hydroponic containers in the greenhouse (Unifarm, Wageningen University & Research). Uniform seedlings with four leaves were selected and transferred to the hydroponics system for evaluation. Four independently controlled hydroponics units were used; two units for control and the other two for the salt treatment (Supplemental Figure 1), and each unit consisted of 12 connected containers that could hold 12 plants. The hydroponics system was filled with half-strength modified Hoagland's solution. After 1 week in the hydroponics system, NaCl was added to two of the four units with a 50 mM daily increment to bring the final concentration to 150 mM NaCl. The experiment had a split plot design with four replicate plants per genotype per treatment. For this, the 70 genotypes and two dummy plants were randomly assigned to the plant positions in six containers as one replication. Two replications of 70 genotypes were grown in 12 containers on each unit, to a total of four replications on two units per treatment. The nutrient solution was refreshed weekly and maintained at pH 5.8. The greenhouse conditions were similar to the pilot experiment.

### Assessment of growth traits

During the experiment, data was collected for plant height, leaf expansion, and tiller number for all plants grown under control and saline conditions. Plant height was measured from the base of the plant to the tip of the highest leaf with a ruler at day 1, day 10 and day 17 after starting the stress treatment. Growth rate was taken as the growth in height per day, expressed as cm/day. This was calculated as the difference in plant height between two timepoints, divided by the number of days between the timepoints. To measure leaf expansion, the youngest leaf of each plant was marked at the beginning of salt treatment and the length of this leaf was measured three times, 1, 3, 5, and 7 days after starting the stress treatment. Leaf expansion rate was expressed as the average leaf length increase per day and calculated as the difference of the leaf lengths at day 7 and day 1 divided by the number of days between these measurements (expressed as cm/day). Leaf senescence was measured by visual scoring of all leaves of each plant 17 days after starting the salt treatment. Leaf senescence scale is from 1 to 9 according the percentage of senescence area (1 = no senescence, 3 = senesced area 1–30%, 5 = senesced area 30–60%, 7 = senesced areas 60–90%, 9 = senesced area >90%). At harvest, 17 days afer starting the stress treatment, all seedlings from the control and salt treatments were separated into shoots and roots. Plant shoot fresh weight was measured immediately at harvest. Both plant parts were dried separately in a forced-air oven at 70°C for 2 days, and the dry weight was measured.

### Ion chromatography

For determination of the ion concentrations in the shoots and roots of each genotype, four replicated samples per genotype were ground to fine powder using a hammer mill with 1 mm sieve following the protocol described by Nguyen et al. (2013). Dry leaf and root powders (25 ± 1 mg) were ashed at 575°C for 5 h. Ashed samples were dissolved by shaking for 30 min in 1 ml 3 M formic acid at 99°C and then diluted with 9 ml MiliQ water. The samples were shaken again at 80°C for another 30 min. A final 500x dilution was subsequently prepared by mixing 0.2 ml sample solution with 9.8 ml MiliQ to assess the Na^+^, K^+^, Cl^−^, and Ca^2+^ content of each root and leaf sample using Ion Chromatography (IC) system 850 Professional, Metrohm (Switzerland).

### Statistical analysis of phenotypic data

Analysis of variance (ANOVA) was done in a split plot design using Genstat 15th version. The four hydroponics units contained four replicated whole plots (schematically represented in supplementary Figure 1). The whole plots were divided in two split plots of two adjacent units. The two treatments were assigned to one of the two units in a split plot. Each split plot contained six adjacent containers as a block (2 blocks per unit, and four blocks per treatment). Within each block, genotypes were randomly distributed. The growth rate and leaf expansion of each genotype in control and saline conditions were compared by student's *T*-test. Correlation coefficients (r) among all the parameters were calculated. All statistical analyses were performed using the statistical software package Genstat 15th edition (VSN International Hemel Hempstead, UK).

## Results

### Growth responses to salinity stress

In a pilot experiment, two genotypes (OPM-13 and OPM-38) were grown on hydroponics at three different salt conditions (0, 125, and 250 mM NaCl). Growth of these *Miscanthus* genotypes was already affected at 125 mM (Shoot Dry Weight was reduced by 24 and 68% for OPM-38 and OPM-13, respectively, and 36 and 63% at 250 mM NaCl). At both salinity levels, Na^+^ and Cl^−^ concentrations of the shoots were significantly increased (Supplementary Table 2). The high salt concentration of 250 mM seriously damaged the seedlings, which may confound the physiological interpretation of ion concentration data in relation to ion homeostasis. We concluded that a salt stress of 150 mM NaCl of the plants would affect growth of the plants considerably but inflict only limited damage. Therefore, we chose a salt stress level of 150 mM NaCl for identifying salt tolerant genotypes and traits contributing to salt tolerance.

**Figure 1:**
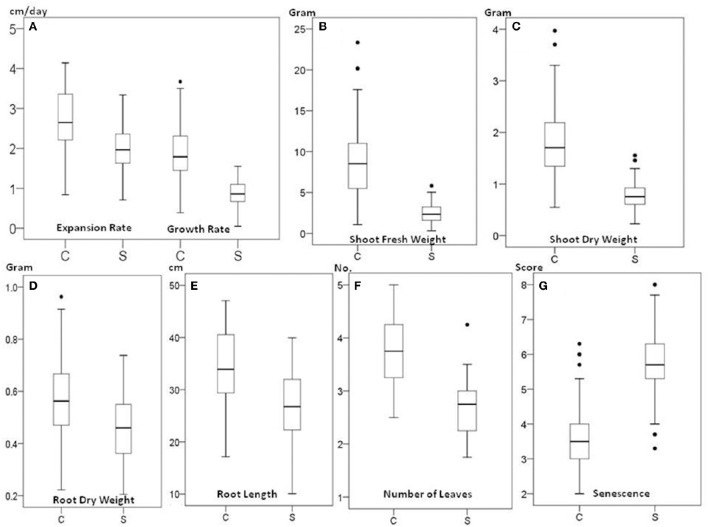
**Box plots of growth trait data of *Miscanthus* under 0 mM NaCl (C) and 150 mM NaCl (S)**. Expansion and growth rate **(A)**, shoot fresh weight **(B)**, shoot dry weight **(C)**, root dry weight **(D)**, root length **(E)**, number of leaves **(F)**, and senescence **(G)**. Box edges show upper and lower quartile and the median is shown in the middle of the box. Mild outliers are shown as dots.

The 70 genotypes showed a wide variation in response to 150 mM NaCl salt treatment. There were significant differences in leaf expansion, growth rate, shoot fresh weight, shoot dry weight, root dry weight, root length, the number of leaves, and senescence score between the 70 genotypes (*P* < 0.001) and between control and salt treatment (*P* < 0.001) (Figures 1A–G). The reduction under saline conditions compared to control conditions for expansion of young leaves and growth rate in plant height was 27 and 54%, respectively. The average shoot dry weight decreased by 58% from 1.83 g under control conditions to 0.77 g under salt stress conditions. The average root dry weight was also decreased but to a lesser extent, from 0.57 g in control conditions to 0.45 g under salt stress. The average number of leaves was reduced from 3.8 to 2.6 as a result of salt stress, and senescence was increased around 1.5-fold at harvest in salt-stressed plants.

### Growth rates

The height of the salt treated plants was reduced 14–88% while the growth rate was decreased from 41 to 86% in the 70 genotypes. The growth rate of the seedlings was highly correlated to height both under salt (*r* = 0.81) and control conditions (*r* = 0.94). This trait also showed significant correlation with shoot dry weight under salinity (*r* = 0.68) and control conditions (*r* = 0.76). The growth rate of 22 genotypes was not significantly different at early stages between control and salt conditions (Table 2).

**Table 2:** **Plant growth rate (plant height increase) and leaf expansion rate of leaves of miscanthus genotypes grown on hydroponics at 0 mM NaCl and 150 mM NaCl**.

**Genotype**	**Growth rate (cm/day)**				**Sig**.	**Expansion rate (cm/day)**				**Sig**.
	**0 mM**		**150 mM**			**0 mM**		**150 mM**		
	**Mean**	**S.D**.	**Mean**	**S.D**.		**Mean**	**S.D**.	**Mean(cm)**	**S.D**.	
OPM-4	2.31	1.08	0.56	0.38	^*^	3.39	0.62	2.38	0.71	
OPM-5	2.98	0.36	1.38	0.31	^***^	3.66	0.69	2.92	0.76	
OPM-6	2.56	0.43	0.68	0.26	^***^	3.91	1.34	2.06	0.15	^*^
OPM-7	2.00	0.57	1.43	0.22		2.05	0.76	2.47	0.23	
OPM-8	2.29	0.45	0.53	0.25	^***^	2.46	0.95	1.54	0.24	
OPM-9	1.77	0.80	0.57	0.39	^*^	1.87	0.80	1.64	0.57	
OPM-10	1.51	1.13	0.71	0.50		3.28	1.29	2.36	0.95	
OPM-11	2.52	0.36	1.55	0.34	^**^	3.95	0.54	3.13	0.52	
OPM-16	1.99	0.89	0.90	0.43		3.62	0.93	2.23	0.40	^*^
OPM-19	3.50	0.78	1.36	0.25	^**^	4.14	0.19	2.59	0.24	^***^
OPM-20	2.67	0.72	1.06	0.95	^*^	3.89	0.61	2.67	0.61	^*^
OPM-24	3.10	1.27	0.78	0.47	^*^	3.36	1.01	2.00	1.07	
OPM-26	1.40	0.80	0.38	0.39		1.52	0.35	1.93	0.64	
OPM-30	1.81	0.52	0.86	0.47	^*^	2.65	0.58	1.76	0.15	^*^
OPM-31	1.14	0.32	0.60	0.18	^*^	1.94	0.71	1.67	0.44	
OPM-32	3.67	0.41	1.28	0.48	^***^	3.68	0.97	2.78	1.00	
OPM-33	1.11	0.31	0.42	0.12	^**^	1.53	0.69	0.99	0.27	
OPM-34	1.28	0.55	0.75	0.12		1.34	0.39	1.20	0.30	
OPM-37	2.27	1.00	0.95	0.52		2.82	1.28	2.37	0.70	
OPM-41	1.47	0.28	0.71	0.33	^*^	2.44	0.11	1.51	0.44	^**^
OPM-42	1.49	0.50	0.91	0.17		2.35	0.65	2.01	0.33	
OPM-44	0.81	0.68	0.40	0.30		1.81	0.49	1.65	0.31	
OPM-45	1.47	0.57	0.38	0.34	^*^	1.30	0.71	0.95	0.28	
OPM-47	0.39	0.16	0.05	0.03	^**^	0.84	0.28	0.71	0.08	
OPM-48	1.78	0.12	1.08	0.26	^**^	2.35	0.23	2.40	0.34	
OPM-49	1.87	0.52	0.82	0.19	^**^	3.14	0.44	2.06	0.15	^**^
OPM-50	1.44	0.39	0.58	0.49	^*^	3.68	1.01	2.49	0.18	
OPM-56	2.24	0.30	0.89	0.84	^*^	3.45	0.67	2.46	0.74	
OPM-57	3.02	0.91	0.88	0.18	^**^	4.10	0.51	2.39	0.27	^***^
OPM-58	1.57	0.52	0.61	0.46	^*^	2.66	0.50	1.41	0.54	^*^
OPM-59	2.50	0.05	1.17	0.14	^***^	3.56	0.25	2.41	0.20	^***^
OPM-62	1.54	0.42	0.46	0.34	^**^	2.75	0.59	1.49	0.37	^*^
OPM-64	1.61	0.30	1.07	0.34		2.66	0.24	1.97	0.41	^*^
OPM-65	2.35	0.36	1.22	0.27	^**^	3.13	0.37	2.10	0.18	^**^
OPM-66	0.96	0.12	0.82	0.14		1.54	0.08	1.49	0.27	
OPM-67	1.23	0.63	0.47	0.38		2.67	0.86	1.89	0.19	
OPM-68	0.84	0.62	0.59	0.13		1.49	0.44	1.31	0.25	
OPM-69	2.76	0.68	1.28	0.50	^*^	2.31	0.94	2.12	0.50	
OPM-71	2.00	0.84	1.31	0.20		3.00	0.72	2.46	0.15	
OPM-72	1.45	0.62	1.01	0.35		2.42	0.67	1.84	0.09	
OPM-73	1.61	0.92	0.90	0.41		2.22	1.38	2.54	0.65	
OPM-74	2.24	0.26	0.86	0.46	^**^	2.58	0.70	1.81	0.69	
OPM-75	1.65	0.17	1.11	0.24	^**^	2.32	0.36	1.93	0.25	
OPM-76	1.24	0.87	0.72	0.26		2.23	0.41	2.08	0.36	
OPM-77	1.83	0.45	1.00	0.49	^*^	2.21	0.39	1.50	0.32	^*^
OPM-78	2.25	0.33	1.27	0.33	^**^	2.70	0.93	2.13	0.81	
OPM-79	3.39	0.51	1.21	0.84	^**^	4.00	1.01	3.34	0.83	
OPM-81	1.42	0.39	0.68	0.20	^*^	2.22	0.71	1.43	0.35	
OPM-82	1.58	0.53	1.10	0.11		2.65	0.22	2.09	0.26	^*^
OPM-83	1.35	0.48	0.88	0.16		1.89	0.56	1.63	0.16	
OPM-84	1.96	0.36	1.07	0.20	^**^	2.85	0.41	2.31	0.12	^*^
OPM-86	1.80	0.20	0.83	0.28	^***^	2.39	0.61	1.81	0.39	
OPM-87	2.50	0.20	0.83	0.44	^***^	3.26	0.27	1.96	0.50	^**^
OPM-88	2.10	0.86	0.89	0.34	^*^	2.63	1.21	1.91	0.53	
OPM-89	2.48	0.14	1.15	0.08	^***^	3.65	0.24	2.07	0.39	^***^
OPM-90	1.80	0.22	0.78	0.05	^***^	2.88	0.38	1.96	0.10	^**^
OPM-91	1.78	0.45	0.74	0.38	^*^	2.82	0.57	1.69	0.48	^*^
OPM-92	2.70	0.29	1.46	0.34	^***^	3.74	0.38	2.35	0.16	^***^
OPM-94	0.88	0.42	0.50	0.38		0.94	0.58	0.87	0.30	
OPM-96	2.08	0.32	0.76	0.41	^**^	3.29	0.27	1.72	0.52	^**^
OPM-97	1.96	0.40	1.22	0.16	^*^	3.34	0.24	2.17	0.30	^***^
OPM-98	1.61	0.48	1.06	0.47		2.24	0.82	1.84	0.25	
OPM-99	1.58	0.21	0.89	0.10	^***^	2.65	0.39	2.25	0.75	
OPM-100	1.76	0.40	0.78	0.49	^*^	1.61	0.56	1.37	0.41	
OPM-13	1.17	0.64	0.61	0.44		2.18	0.32	1.62	0.39	
OPM-103	1.11	0.35	0.53	0.46		1.56	0.43	1.20	0.24	
OPM-104	1.45	0.38	0.67	0.30	^*^	2.21	0.46	1.70	0.49	
OPM-107	3.00	0.27	1.14	0.36	^***^	3.78	0.36	2.24	0.32	^***^
OPM-108	1.42	0.28	0.85	0.25	^*^	1.80	0.32	1.39	0.14	
OPM-109	2.90	0.59	1.54	0.47	^*^	3.51	0.95	2.41	0.59	

*, **, *** *, significant at P < 0.05; 0.01; 0.001 respectively*.

### Leaf expansion rates

The leaf expansion rate of the 70 genotypes was on average reduced by 27% from 2.67 cm/day under control conditions to 1.96 cm/day under saline conditions. Expansion rate of the second young leaf showed a more marked difference between the salt-treated and control seedlings than the flag leaf. There were significant effects for genotype (*p* < 0.001), treatment (*p* < 0.001) and genotype by treatment interaction (*p* = 0.004) for leaf expansion rate (Table 2). Expansion rate differences between control and salt-treated genotypes ranged from 3 to 48%. In 46 genotypes, the leaf expansion under salt stress was not significantly different from control. Leaf expansion rate significantly correlated with shoot dry weight under salinity (*r* = 0.86) and control conditions (*r* = 0.82).

### Na^+^ accumulation in leaves

**Figure 2:**
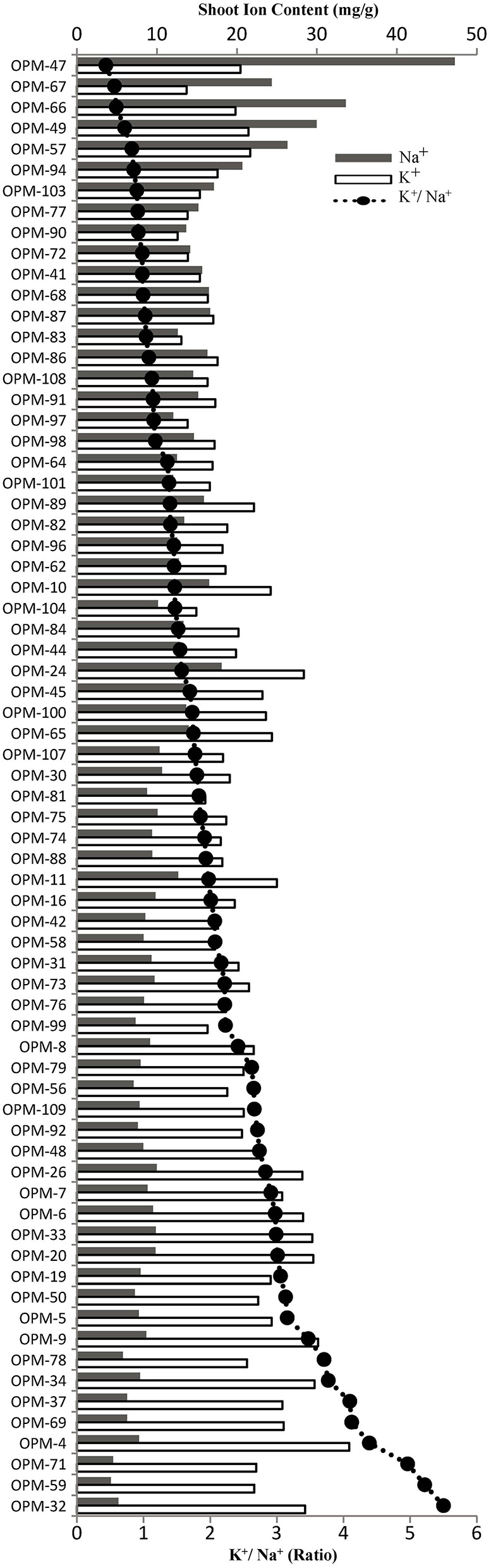
**Shoot Na^+^ and K^+^ concentration (gray and white bars, respectively) and shoot K^+^/Na^+^ ratio (line-scatter plot) in leaves of 70 *Miscanthus* genotypes grown under saline conditions (150 mM NaCl)**.

The 70 genotypes showed large differences in leaf Na^+^ concentration of salt-stressed plants, from 4.25 mg/g in OPM-59 to 47.22 mg/g in OPM-47, and the K^+^/Na^+^ ratio ranged from 5.39 in OPM-59 to 0.49 in OPM-47 (Figure 2, Supplementary Figure 2). Of the six genotypes with the highest Na^+^ concentrations in the leaves (OPM-47, 49, 57, 66, 67, and 94), OPM-49 and 57 had a relatively high tiller number and low percentage of dead leaves and OPM-57 had slightly higher than average biomass (Table 3). This indicates that these genotypes may utilize a tissue tolerance mechanism, possibly by accumulation of Na^+^ in vacuoles. On the other hand, some genotypes showed low shoot sodium concentrations under salt stress. Six genotypes (OPM-4, 32, 37, 59, 69, and 71) not only showed the lowest Na^+^ concentration but also had the highest K^+^/Na^+^ ratio in leaves. Additionally, these genotypes demonstrated less senescence on leaves compared with the high-Na^+^ genotypes, relatively high biomass, and low leaf Na^+^ /root Na^+^ ratio. This indicates that these genotypes may utilize a shoot exclusion mechanism under saline conditions. Among these, OPM-37 was relatively tolerant and it also had the highest biomass of all genotypes under saline conditions (Table 4).

**Table 3:** **Trait comparisons of 6 *Miscanthus* genotypes with high leaf Na^+^ ion concentrations under salt stress, grown at 150 mM NaCl on hydroponics**.

**Genotype**	**OPM-47**	**OPM-49**	**OPM-57**	**OPM-66**	**OPM-67**	**OPM-94**	**Average of 70 genotypes**
Tiller number	0	2.25	2	1.25	2.25	0	1.73
Dead leaves (%)	50	32	35	40	40	56	28
Leaf Na^+^ (mg/g)	47.22	30.36	26.32	33.62	24.35	20.66	12.55
Root Na^+^ (mg/g)	56.5	32.85	33.33	50.99	34.58	29.81	37.23
Leaf K^+^ (mg/g)	20.45	21.44	21.66	19.81	13.69	17.58	20.67
Biomass (g)	0.23	0.63	0.82	0.35	0.64	0.31	0.77
Salt tolerance (%)	41	44	29	41	54	50	43
K^+^/Na^+^ in leaf	0.43	0.71	0.82	0.59	0.56	0.85	2.08
Leaf Na^+^/Root Na^+^	0.84	0.92	0.79	0.66	0.70	0.69	0.34

**Table 4:** **Trait comparisons of 6 *Miscanthus* genotypes with low leaf Na^+^ concentrations under salt stress, grown at 150mM NaCl on hydroponics**.

**Genotype**	**OPM-4**	**OPM-32**	**OPM-37**	**OPM-59**	**OPM-69**	**OPM-71**	**Average of 70 genotypes**
Tiller number	2.8	1	3	1.3	1.8	0	1.7
Dead leaves (%)	29	20	23	22	20	24	28
Leaf Na^+^ (mg/g)	7.76	5.19	6.27	4.25	6.27	4.52	12.55
Root Na^+^ (mg/g)	40.4	37.64	30.04	39	43	42.45	37.23
Leaf K^+^(mg/g)	34.05	28.53	25.68	22.17	25.85	22.41	20.67
Biomass (g)	0.79	1.13	1.56	0.96	0.88	1.05	0.77
Salt tolerance (%)	33	34	49	46	47	46	43
K^+^/Na^+^ in leaf	4.39	5.50	4.01	5.22	4.12	4.96	2.08
Leaf Na^+^/Root Na^+^	0.19	0.14	0.21	0.11	0.15	0.11	0.34

### Ion homeostasis change to salinity stress

**Figure 3:**
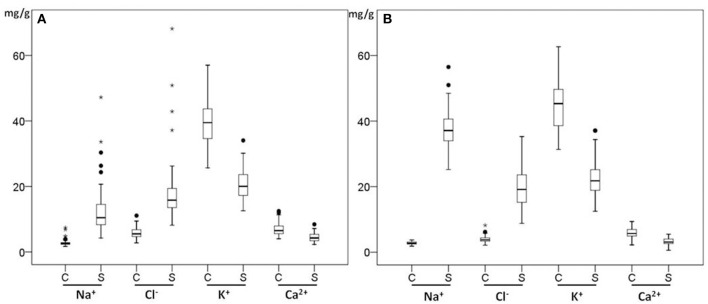
**Box plots of ion concentrations of leaves (A)** and roots **(B)** of 70 *Miscanthus* genotypes in control (C) and salt (S) conditions. Box edges show upper and lower quartile and the median is shown in the middle of the box. Mild outliers are shown as dots and extreme outliers shown as stars.

The boxplots in Figures 3A,B show the genotypic variation of the ion contents in both shoots and roots under control and salt conditions. There were significant differences in the ion concentrations (*P* < 0.001) in shoots and roots of 70 genotypes under control and salt treatment (*P* < 0.001). The interaction between genotypes and treatments was significant (*P* < 0.001) for both Na^+^ and Cl^−^ concentration under salt stress. In both shoots and roots, the Na^+^ and Cl^−^ concentrations increased significantly under salt stress (*P* < 0.001), while [K^+^] and [Ca^2+^] decreased at 150 mM NaCl. In the leaves, Na^+^ and Cl^−^ concentrations increased 4.6- and 3.1-fold under salt treatment, accumulating to 12.55 mg/g for Na^+^ and 18.07 mg/g for Cl^−^ (Figure 3A) but K^+^ and Ca^2+^ concentrations in the shoots under saline conditions were 0.5- and 0.6 -fold lower than those under control conditions. In the roots, Na^+^ and Cl^−^ concentrations showed 13- and 5-fold increases under salt treatment, respectively accumulating to 37.23 mg/g for Na^+^ and 19.66 mg/g for Cl^−^ (Figure 3B) while both K^+^ and Ca^2+^ concentrations decreased by 50% compared with those under control conditions. Under salt stress, Na^+^ concentration in the roots was much higher than in shoots (3.6-fold), while Cl^−^ concentration in roots was slightly higher than in shoots (1.23-fold). This indicates that these genotypes may have an active mechanism to keep the Na^+^ concentration low in the shoots.

### Salt tolerant genotypes

**Figure 4:**
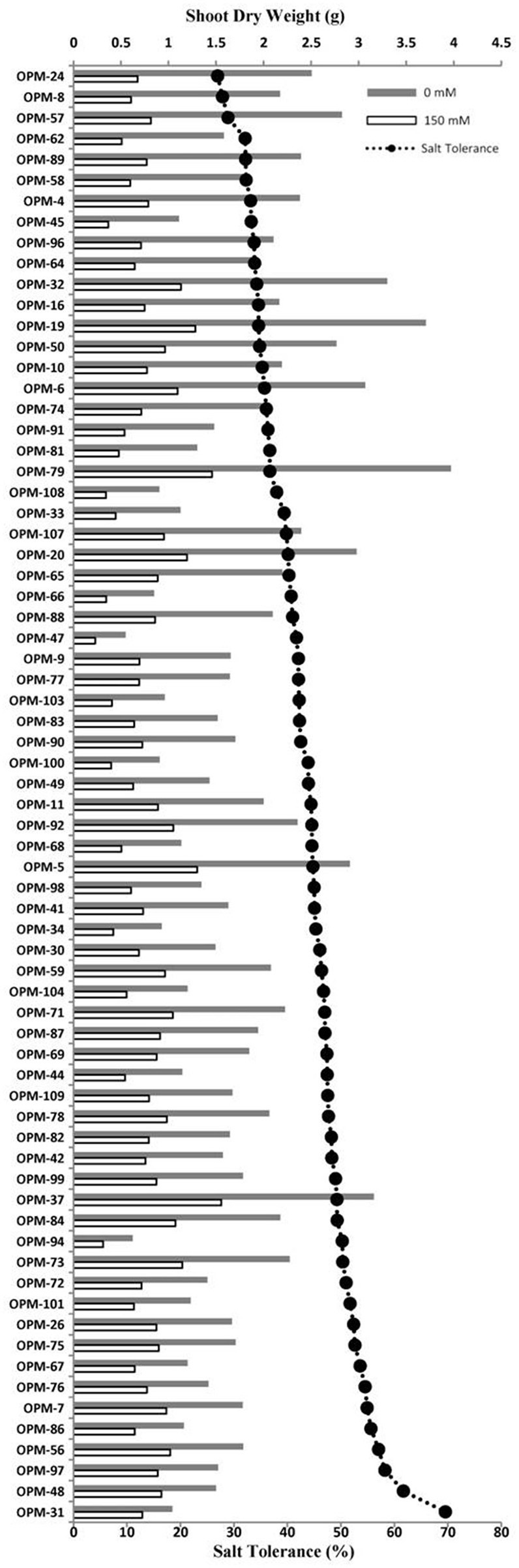
**Shoot dry weight (bars) and salt tolerance (line-scatter plot, calculated as the ratio of shoot dry weight under salt stress and shoot dry weight under control conditions) of 70 genotypes of *Miscanthus* grown in a hydroponics system at 0 mM NaCl (gray bars) and 150 mM NaCl (white bars)**.

Salt tolerance was assessed as the percentage of shoot dry weight under saline relative to control conditions. The set of 70 genotypes grown at 150 mM NaCl in the hydroponic system showed large variation for salt tolerance, from 26% for OPM-24 to 69% for OPM-31 (Figure 4, Supplementary Figure 3). Salt tolerance of the commercial genotype OPM-9 (*Miscanthus* × *giganteus*) was 42%. The shoot dry weight in salt stress varied from 0.23 to 1.56 g, and from 0.55 to 3.97 g under control conditions. The reduction in shoot dry weight ranged from 30 to 73%. It is interesting to note that the genotypes with high salt tolerance (over 50%, <50% reduction in biomass) generally had relatively low biomass under control conditions. The top 10 genotypes for salt tolerance had less biomass (1.48 g) compared to overall average (1.83 g) under control conditions but the biomass was slightly higher than average (0.84 vs. 0.77 g) under salt stress (Supplementary Table 3). Those genotypes therefore were the most tolerant, but typically not the most productive under control conditions. The top 10 genotypes with high yield had on average more biomass under control conditions (2.99 vs. 1.83 g) and more biomass compared to the overall average under salt stress (1.23 vs. 0.77 g). These genotypes were still more productive under saline conditions, even if they were less tolerant to salinity (Supplementary Table 3). The genotype OPM-37 seemed to be interesting because it has the highest yield (1.56 g) under salt stress, is among the higher producers (3.16 g) under control conditions, and is relatively salt tolerant (49%).

### Associations between growth traits and salt

Correlations between the different physiological traits and ion concentrations are given in Table 5. A highly significant negative correlation of Cl^−^ and Na^+^ concentrations in shoots was found with growth traits (shoot dry weight, shoot fresh weight, root dry weight, and root length) under salt stress. Leaf Cl^−^ and Na^+^ concentrations were negatively correlated (*P* < 0.001) to the shoot biomass (*r* = −0.43 and −0.53, respectively) at 150 mM NaCl. Under salt treatment, there was a high correlation between Cl^−^ and Na^+^ concentrations in both leaves (*r* = 0.94) and roots (*r* = 0.66) but under control conditions there was only a weak correlation in leaves (*r* = 0.26) and no significant correlation in roots. The shoot dry weight was positively correlated with leaf expansion rate, root dry weight, growth rate, and root length under salt stress (*r* = 0.86, 0.85, 0.68, and 0.62, respectively). The correlation between K^+^ and Na^+^ concentrations in leaves and roots were not significant under salt stress while there was weak correlation for these traits in both leaves (*r* = 0.48) and roots (*r* = 0.44) under control conditions. However, the K^+^ concentration in leaves was positively correlated with shoot fresh weight (*r* = 0.41) and weakly correlated with shoot dry weight (*r* = 0.30) at 150 mM NaCl, similar to the correlations at 0 mM NaCl (*r* = 0.4 and 0.28, respectively). The ratio of K^+^/Na^+^ was positively (*P* < 0.001) related to the shoot biomass (*r* = 0.56) in all genotypes under salt treatments but it was weak (*r* = 0.31) under control conditions.

**Table 5:**
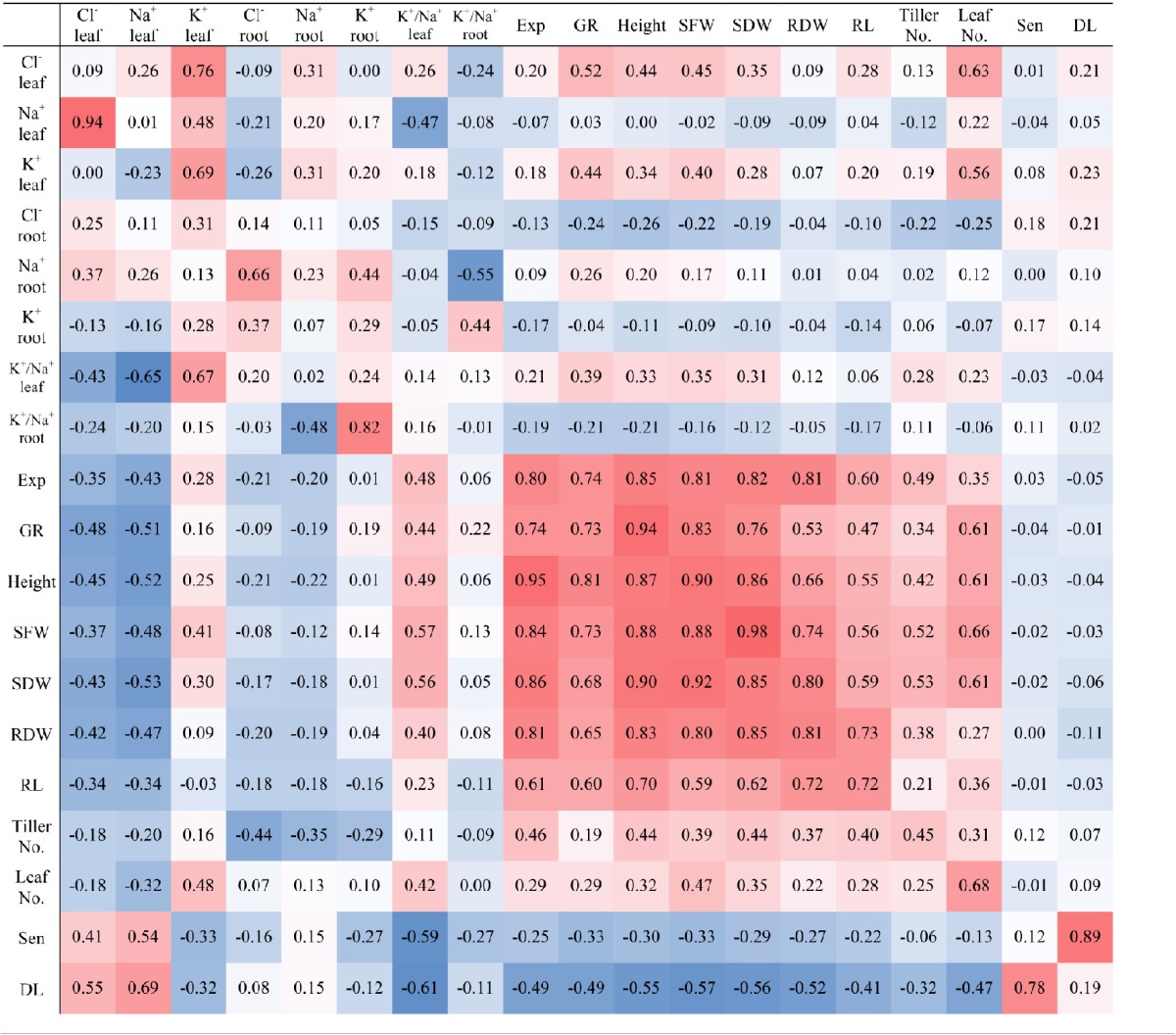
**Pearson correlations between the traits under salt stress (left lower triangle) and control (right upper triangle)**.

*The left upper to right lower corner diagonal indicates the correlation between trait values for control and saline conditions*.

*Sen, Senescence; DL, Dead leaves; Exp, Expansion rate; GR, Growth rate; SFW, Shoot Fresh Weight; SDW, shoot dry weight; RDW, Root dry weight; RL, Root length*.

*From light red to dark red, increasingly more positive correlation. From light blue to dark blue, increasingly more negative correlation*.

## Discussion

Bioenergy crops are an important alternative to fossil fuel, and a valuable addition to other alternative forms of energy (Brosse et al., 2012). Growing these crops on underutilized, marginal soils like saline soils would avoid competition with food crops for agricultural land. The potential for improvement of *Miscanthus* for salinity tolerance still remains to be established, as most research has focused only on *Miscanthus* × *giganteus* (Plazek et al., 2014; Stavridou et al., 2016) and genetic diversity for salinity tolerance of *Miscanthus* germplasm is largely unknown. The current study evaluated seventy *Miscanthus* genotypes under salt stress and showed that broad diversity for salt tolerance and salt tolerance traits is present in *Miscanthus*. Several highly salt tolerant genotypes utilizing different mechanisms can be considered as valuable breeding material.

### Screening system

A reliable screening system for salt tolerance traits is essential, as uniform exposure of plants to salt stress is hard to establish and control in field experiments (Munns and James, 2003; Almeida et al., 2016). Hydroponic systems supply uniform conditions for the root environment, and have a high capacity of genotypes at the same time (Nguyen et al., 2013; Chan-Navarrete et al., 2014). Using such a system, traits, and QTLs contributing to variation in salt tolerance in barley were already successfully identified (Long et al., 2013; Nguyen et al., 2013), and to variation in nitrogen use efficiency in spinach (Chan-Navarrete et al., 2016). It is important to keep in mind however that factors like soil texture and composition that in the field also may influence salinity tolerance do not play a role in this type of system. Also, root properties related to soil traits and exploration of the soil will have a different impact on growth and yield. Another limitation of hydroponics evaluation is that it only allows screening of relatively young plants. Nevertheless, given the difficulty to maintain uniform screening conditions in a large population in the field (Tavakkoli et al., 2012), hydroponics provides a highly useful alternative. It is a fast and uniform way to identify high potential genotypes with interesting salt tolerance traits that particularly relate to ion homeostasis and other cellular tolerance mechanisms, like osmotic adjustment and scavenging of reactive oxygen species (ROS). Indeed, several studies on salt tolerance using hydroponics systems found correlations between salt tolerance and Na^+^ and K^+^ concentrations in shoot (Munns and James, 2003; Jaarsma et al., 2013; Platten et al., 2013). Similarly, we identified several salt tolerant genotypes in our hydroponics-based screening with low Na^+^ concentrations in the leaves (Table 4). These are likely to utilize Na^+^ exclusion mechanisms and may be useful genitors for salinity tolerance breeding programs. OPM-37 was even among the highest biomass producers both under control and salt conditions, and should be evaluated under field conditions as a potential high producing genotype on saline soils.

### Mechanisms and useful traits

When grown in saline soils, plants are exposed to osmotic stress and ionic stress (Ashraf and Harris, 2004; Munns and Tester, 2008). Since osmotic and ionic stress both decrease yield and growth rate, improving salt tolerance in crops needs to take into account both osmotic tolerance and ion exclusion (Genc et al., 2010). Osmotic tolerance appears to contribute more to salt tolerance than avoiding ion toxicity in cultivated wheat and in barley (Rengasamy, 2010a). Leaf expansion is considered a good indicator for osmotic tolerance (Rajendran et al., 2009; Farouk, 2011). In our tested *Miscanthus* genotypes, leaf expansion showed highly significant correlation with shoot dry weight (*r* = 0.86) under salinity. The relatively high variation in the leaf expansion measurements may be caused by the relatively high variation in youngest leaf length between replicates of a genotype at the start of the measurements. This may be avoided by using more replicate plants and selecting only the plants with youngest leaves of comparable lengths, but that would require either a higher capacity (number of plants) of plants, or a reduction in the number of genotypes.

The most likely candidate genotypes to have osmotic tolerance may be the ones that have both limited reduction in both leaf expansion rate and in growth rate at early stages of the stress. Forty-six of the *Miscanthus* genotypes evaluated in this study showed no significant difference in expansion rate and 22 genotypes had no significant difference in growth rate as a result of salt stress (Table 2). Nineteen genotypes (OPM-7, 10, 26, 34, 37, 42, 44, 66, 67, 68, 71, 72, 73, 76, 83, 94, 98, 13, and 103) had both little reduction of leaf expansion and height, which would imply that more than 25% of the tested genotypes may have some level of osmotic tolerance that minimizes the early effects of salinity.

Ion toxicity is induced by prolonged salinity due to the accumulation of ions in plant tissues, especially in the leaves (Munns and James, 2003). The leaf blades are more sensitive to ion toxicity than the roots, so adapting ion homeostasis to keep a relatively low Na^+^ concentration in the leaf is important (Munns and Tester, 2008). Under salt stress, high Na^+^ concentrations interfere with K^+^ uptake and K^+^ function (Shabala and Cuin, 2008). Maintaining a high K^+^ concentration at relatively high Na^+^ levels is therefore another important mechanism under salt stress, and the K^+^/Na^+^ ratio is considered an indicator of salt tolerance (Munns and James, 2003; Krishnamurthy et al., 2007). In a large-scale screen of69 barley cultivars, 90% of the genotypes used an active K^+^ maintenance mechanism to retain cytosolic K^+^ concentrations, while 10% achieved this indirectly by efficiently excluding Na^+^ from shoot (Chen et al., 2007; Schmer et al., 2008). In our evaluation, the K^+^/Na^+^ ratio was not only positively correlated (*r* = 0.56) to shoot dry weight in the 70 tested genotypes, but also negatively correlated (*r* = −0.59) to senescence under salt stress. Only 12 of the 70 genotypes had a K^+^/Na^+^ ratio of <1 (Figure 2), indicating that most *Miscanthus* genotypes were able to maintain a relatively high K^+^ concentration compared to the Na^+^ concentration.

Six genotypes with a high K^+^/Na^+^ ratio (more than twice the average value) had low Na^+^ concentrations in the leaves (Table 4). Low Na^+^ concentration in the shoots was successfully used as selection criteria to breed for salt tolerant cultivars in wheat, barley and rice (Lin et al., 2004; Lindsay et al., 2004; Xue et al., 2009; Genc et al., 2010; Thomson et al., 2010; Munns et al., 2012). We identified a number of genotypes with low Na^+^ concentrations in the shoot and high salt tolerance, suggesting that these utilize Na^+^ exclusion mechanisms similar to those used for improving salt tolerance in cereals. The genotypes with the lowest Na^+^ concentrations in leaves also showed the lowest Na^+^ shoot/ Na^+^ root ratio (Table 4) implying that Na^+^ is actively excluded from the shoots. The gene underlying the Na^+^ exclusion introduced from wild relatives in both rice and wheat was shown to be a member of the Na^+^-selective transporter HKT gene family. This HKT1;5 gene is expressed in parenchyma cells aligning the xylem in roots, and the HKT1;5 transporter was shown to filter Na^+^ out of the xylem, thus preventing transport Na^+^ from the roots to the shoots (Maser et al., 2002; Husain et al., 2003). Seven major and three minor alleles of OsHKT1;5 were identified in rice and the leaf Na^+^ concentration was highly associated with *HKT1;5* allelic variation across diverse accessions (Platten et al., 2013). It is not unlikely that a *Miscanthus* HKT1;5 ortholog is responsible for the variation in Na^+^ shoot concentration in *Miscanthus*. It would therefore be worthwhile to study allelic variation and activity of this *Miscanthus* HKT1;5 ortholog under saline conditions in *Miscanthus* genotypes.

Because electrochemical balance is vital under stress, Cl^−^ and Na^+^ uptake are often linked (Teakle and Tyerman, 2010). However, the Na^+^ and Cl^−^ exclusion mechanisms are independent, with different genotypes having different mechanisms to regulate Na^+^ or Cl^−^ transport (Teakle and Tyerman, 2010). For example, genotypes of *Glycine max* were more sensitive to Cl^−^ ion accumulation, but *G. soja* genotypes were more sensitive to high levels of Na^+^ ions (Luo et al., 2005). In our *Miscanthus* genotypes, the average Cl^−^ root/shoot ratio was 1.23 but the Na^+^ root/shoot ratio was 3.6 under salt treatment over 70 genotypes (Figure 3). This indicates that an active mechanism to avoid accumulation of Na^+^ in the leaves is relatively abundant in *Miscanthus*, and a similar mechanism for Cl^−^ ion accumulation in the shoots is much less prevalent. Nevertheless, there was a high correlation (*r* = 0.94) between Cl^−^ and Na^+^ concentrations in leaves and both Cl^−^ and Na^+^ had negative correlations with shoot dry weight stress, *r* = −0.43 and *r* = −0.53 respectively (Table 5). It is interesting that four genotypes (OPM-59, 71, 78, and 109) showed low Cl^−^ concentrations (8.14–10.09 mg/g) compared with the average (18.06 mg/g) in leaves as well as a relatively high Cl^−^ root/shoot ratio (0.41–0.49) compared with the average (0.98). Those genotypes may have Cl^−^ exclusion mechanisms (Supplementary Table 4).

In the present study, two genotypes (OPM-49 and 57) also showed more tillers and less senescence even with having high shoot concentrations of Na^+^ and Cl^−^. This may be indicative for a tissue tolerance mechanism, with Na^+^ and Cl^−^ compartmentalized into the vacuoles to avoid toxic concentrations within the cytoplasm (Munns and James, 2003).

### Rhizome

Root traits studied in a hydroponic system may not be representative for root characteristics in the soil and the effect these have on yield (Tavakkoli et al., 2012). For a perennial with a rhizome, like *Miscanthus*, this may be even more true. Chinese ryegrass *Leymus chinensis* can adapt to salt stress by accumulating Na^+^ in the rhizome (Mann et al., 2013; Li et al., 2014). A similar result was found for *Miscanthus* × *giganteus* in a pot experiment; the Na^+^ concentration in rhizomes was 3-fold higher in the rhizome than that in shoot under 150 mM NaCl, and plants with larger rhizomes were more tolerant than plants with small rhizomes, with lower decreases in shoot dry weight under salinity (Plazek et al., 2014). This indicates that rhizomes may play an important role in salt tolerance of perennial grasses, and obviously this component of salt tolerance cannot be tested on a hydroponics system. However, keeping the limitations of the hydroponics system in mind, the advantages in terms of uniformity of plants and environmental conditions, as discussed before, can be exploited. We have shown here that identification of genetic variation for salt tolerance traits, and of mechanisms utilized by *Miscanthus* to counteract the effect of salinity can be done effectively on a hydroponics system. A selection of genotypes with varying salt tolerance properties could thus be made, and these can be used to study salt tolerance mechanisms in more detail in soil-grown plants in pots or in the field.

### Preferred genotypes

Although *Miscanthus* × *giganteus* with its high yield is the most popular commercial genotype, it has several disadvantages. Firstly, its tolerance to abiotic stress is not as high as *M. sinensis* (Clifton-Brown et al., 2001). With respect to chilling tolerance, the rhizomes of *Miscanthus* × *giganteus* cannot survive below approximately −3°C but the hybrids of *M. sinensis* still live below −4.5°C (Clifton-Brown and Lewandowski, 2000). Shoot dry weight of *Miscanthus* × *giganteus* in pots was reduced by 50% after 64 days at 120 mM NaCl (Stavridou et al., 2016), while *M*. *sinensis* accessions exhibited <40% reduction under the same levels of salt stress (Sun et al., 2014). In our experiment, the reduction of *Miscanthus* × *giganteus* (OPM-9) at 150 mM NaCl for 2 weeks was 57%, which was identical to the average salt tolerance in 70 genotypes. This offers opportunities for selecting and breeding genotypes that surpass *Miscanthus* × *giganteus* in salt tolerance and growth on marginal, saline soils. OPM-37 for instance had the highest yield under salt stress, and OPM-31 the lowest reduction compared to yield under control conditions. The hybrids OPM-5 (*M. sinensis* × *M. sacchariflorus*), and OPM-7 (*M. sacchariflorus* × *M. sinensis*) used in our study had higher yield than *Miscanthus* × *giganteus* under control and salt stress as well as higher salt tolerance. These genotypes may be favorable choices for producing biomass on saline lands, and also may indicate the potential of new hybrids that combine favorable traits identified in this study. Lewandowski et al. (2016) indicated that OPM-5 and OPM-7 in a multi-location trial were not among the highest biomass producers under non-saline conditions. Whether these genotypes will be relatively good performers on saline soils remains to be established. Several genotypes had relatively high yields under both control and saline conditions, and may be preferred in soils with varying levels of salinity. These include OPM-5, OPM-6, OPM-19, OPM-20, OPM-32, OPM-37, and OPM-79. Among these seven genotypes, OPM-37, and OPM-5 have salt tolerance of 49% and 44%, respectively, just above the average (43%). These two genotypes may show osmotic mechanisms with limited reduction of the expansion rate. OPM-37 and OPM-5 had a less than average reduction in tiller number due to salt stress, and above average tiller number (3 and 3.25 respectively, while average tiller number was 1.7) under salt stress. These two genotypes have relatively high potential to be cultivated on marginal lands.

It is important to take into account how *Miscanthus* quality is used for bioenergy when choosing optimal genotypes for growth under saline conditions, or genitors for breeding programs. A low ion content of harvested material is very important for combustion quality because the high mineral content can reduce the ash melting point and cause corrosion issues, especially K^+^ and Cl^−^ (Brosse et al., 2012). Jorgensen (1997) showed that during harvest season (spring) the K^+^ and Cl^−^ concentrations in *M. sinensis* were reduced by 85–95% in the normal field because of relocation of minerals to storage organs and leaching by rain. However, the potential impact on combustion properties for material grown on saline lands is largely unknown. Whether the ions accumulate in the senesced stem that is harvested still needs to be established. If the Na^+^ and Cl^−^ accumulate in the shed leaves but not in the stems, genotypes with salt inclusion could also be considered as good genitors for breeding. If the ions still accumulate in the stems, the genotypes with salt exclusion would be preferred as starting material for breeding; OPM-59 and OPM-71 would be good candidates, with lowest concentrations of Na^+^ and Cl^−^ under salt stress in the shoots. Another quality aspect to consider is the cell wall; stress is known to cause changes in the cell wall composition (Le Gall et al., 2015). Drought stress reduced the cellulose content but increased the hemicellulosic polysaccharides so that available cell wall polysaccharides were more easily released as fermentable sugars during processing (van der Weijde et al., 2016). However, the interaction between cell wall composition and salt stress in *Miscanthus* is still unexplored.

## Author contributions

CC performed most of the experiments and wrote the paper. HV assisted with design and execution of the trials and analyses, SD performed the pilot trial, CA, KS, and HM created and provided *in vitro* material. RV contributed to the supervision, experimental strategy and discussion of the outcomes and to correcting the final manuscript, and CV supervised the study and contributed to design, analysis, discussions, and writing of the final manuscript.

## Funding

The research has received funding from the European Union consortium OPTIMISC (project ID 289159).

### Conflict of interest statement

The authors declare that the research was conducted in the absence of any commercial or financial relationships that could be construed as a potential conflict of interest.
