# Supplementary material for: Genetic Diversity of Salt Tolerance in Miscanthus
Source: Front Plant Sci. 2017 Feb 14;8:187. doi: 10.3389/fpls.2017.00187 (PMC5306379; doi:10.3389/fpls.2017.00187)
Supplement: Supplementary file 1 [file Table1.DOCX]

## Supplementary Table 1. The composition of half Hoagland solution

| Macro elements | Conc.[mM] | Micro elements | Conc.[μM] |
| --- | --- | --- | --- |
| NH_4_ | 0.5 | Fe | 35 |
| K | 3 | Mn | 11.8 |
| Ca | 2 | Zn | 1.75 |
| Mg | 1 | B | 43.7 |
| NO_3_ | 6 | Cu | 0.125 |
| SO_4_ | 1.25 | Mo | 0.52 |
| P | 1 |  |  |
| Si | 1.48 |  |  |
