# Supplementary material for: Genetic Diversity of Salt Tolerance in Miscanthus
Source: Front Plant Sci. 2017 Feb 14;8:187. doi: 10.3389/fpls.2017.00187 (PMC5306379; doi:10.3389/fpls.2017.00187)
Supplement: Supplementary file 2 [file Table2.DOCX]

## Supplementary Table 2. Shoot Dry Weight, Shoot Na^+^ content and Shoot Cl^-^ content of two genotypes evaluated in the pilot experiment

| Genotype | Treatment | SDW (g) | Shoot Na^+^(mg/g) | Shoot Cl^-^(mg/g) |
| --- | --- | --- | --- | --- |
| OPM-38 | 0 mM | 2.5 ± 0.2 | 2.9 ± 0.9 | 9.4 ± 0.95 |
|  | 125 mM | 1.9 ± 0.2 | 19 ± 3.2 | 21.2 ± 4.3 |
|  | 250 mM | 1.6 ± 0.2 | 30.8 ± 8.9 | 48.8 ±19.6 |
| OPM-13 | 0 mM | 1.9 ± 0.1 | 1.9 ± 2.5 | 7.9 ± 3.0 |
|  | 125 mM | 0.6 ± 0.1 | 8.9 ± 3.0 | 15.8 ± 3.0 |
|  | 250 mM | 0.7 ± 0.1 | 50.9 ± 7.4 | 92.1 ± 17.3 |
