# Supplementary material for: Genetic Diversity of Salt Tolerance in Miscanthus
Source: Front Plant Sci. 2017 Feb 14;8:187. doi: 10.3389/fpls.2017.00187 (PMC5306379; doi:10.3389/fpls.2017.00187)
Supplement: Supplementary file 3 [file Table3.DOCX]

## Supplementary Table 3. Shoot Dry Weight and Salt Tolerance of the ten genotypes with the highest salt tolerance and the highest yield under salt stress

|  | Shoot dry weight (g) | |  |  | Shoot dry weight (g) | |  |
| --- | --- | --- | --- | --- | --- | --- | --- |
| Genotype | 0 mM | 150 mM | Salt Tolerance (%) | Genotype | 0 mM | 150 mM | Salt Tolerance (%) |
| OPM-31 | 1.04 | 0.72 | 70 | OPM-37 | 3.16 | 1.56 | 49 |
| OPM-48 | 1.50 | 0.93 | 62 | OPM-79 | 3.97 | 1.46 | 37 |
| OPM-97 | 1.52 | 0.89 | 58 | OPM-5 | 2.91 | 1.30 | 45 |
| OPM-56 | 1.79 | 1.02 | 57 | OPM-19 | 3.71 | 1.28 | 35 |
| OPM-86 | 1.16 | 0.65 | 56 | OPM-20 | 2.98 | 1.20 | 40 |
| OPM-7 | 1.78 | 0.98 | 55 | OPM-73 | 2.28 | 1.15 | 50 |
| OPM-76 | 1.42 | 0.77 | 55 | OPM-32 | 3.30 | 1.13 | 34 |
| OPM-67 | 1.20 | 0.64 | 54 | OPM-6 | 3.07 | 1.10 | 36 |
| OPM-75 | 1.71 | 0.90 | 53 | OPM-84 | 2.18 | 1.07 | 49 |
| OPM-26 | 1.67 | 0.87 | 52 | OPM-92 | 2.36 | 1.05 | 45 |
| Average | 1.48 | 0.84 | 57 | Average | 2.99 | 1.23 | 42 |
