# Supplementary material for: Genetic Diversity of Salt Tolerance in Miscanthus
Source: Front Plant Sci. 2017 Feb 14;8:187. doi: 10.3389/fpls.2017.00187 (PMC5306379; doi:10.3389/fpls.2017.00187)
Supplement: Supplementary file 4 [file Table4.DOCX]

## Supplementary Table 4. Trait data of four genotypes with the highest Cl^-^ concentrations in leaves under salt stress

| Genotype | OPM-59 | OPM-71 | OPM-78 | OPM-109 | Average of 70 genotypes |
| --- | --- | --- | --- | --- | --- |
| Tiller number | 1.25 | 0 | 1.50 | 1.25 | 1.73 |
| Dead leaves (%) | 22 | 24 | 24 | 24 | 28 |
| Leaf Cl^-^ (mg/g) | 10.09 | 9.32 | 8.85 | 8.14 | 18.06 |
| Root Cl^-^ (mg/g) | 23.74 | 19.94 | 18.15 | 19.90 | 19.66 |
| Leaf K^+^ (mg/g) | 33.17 | 22.41 | 21.26 | 20.84 | 20.67 |
| Biomass (g) | 0.96 | 1.05 | 0.98 | 0.80 | 0.77 |
| Salt tolerance (%) | 46 | 46 | 47 | 47 | 43 |
| Leaf Cl^-^/Root Cl^-^ | 0.43 | 0.47 | 0.49 | 0.41 | 0.98 |

shoot dry weight under salt stress/ shoot dry weight under control conditions) of 70 *Miscanthus* genotypes grown in a hydroponics system at 0 mM NaCl (gray bars) and 150 mM NaCl (white bars). Values are mean (n= 4)
