# Supplementary material for: Genetic Diversity of Salt Tolerance in Miscanthus
Source: Front Plant Sci. 2017 Feb 14;8:187. doi: 10.3389/fpls.2017.00187 (PMC5306379; doi:10.3389/fpls.2017.00187)
Supplement: Supplementary file 5 [file Image1.PDF]

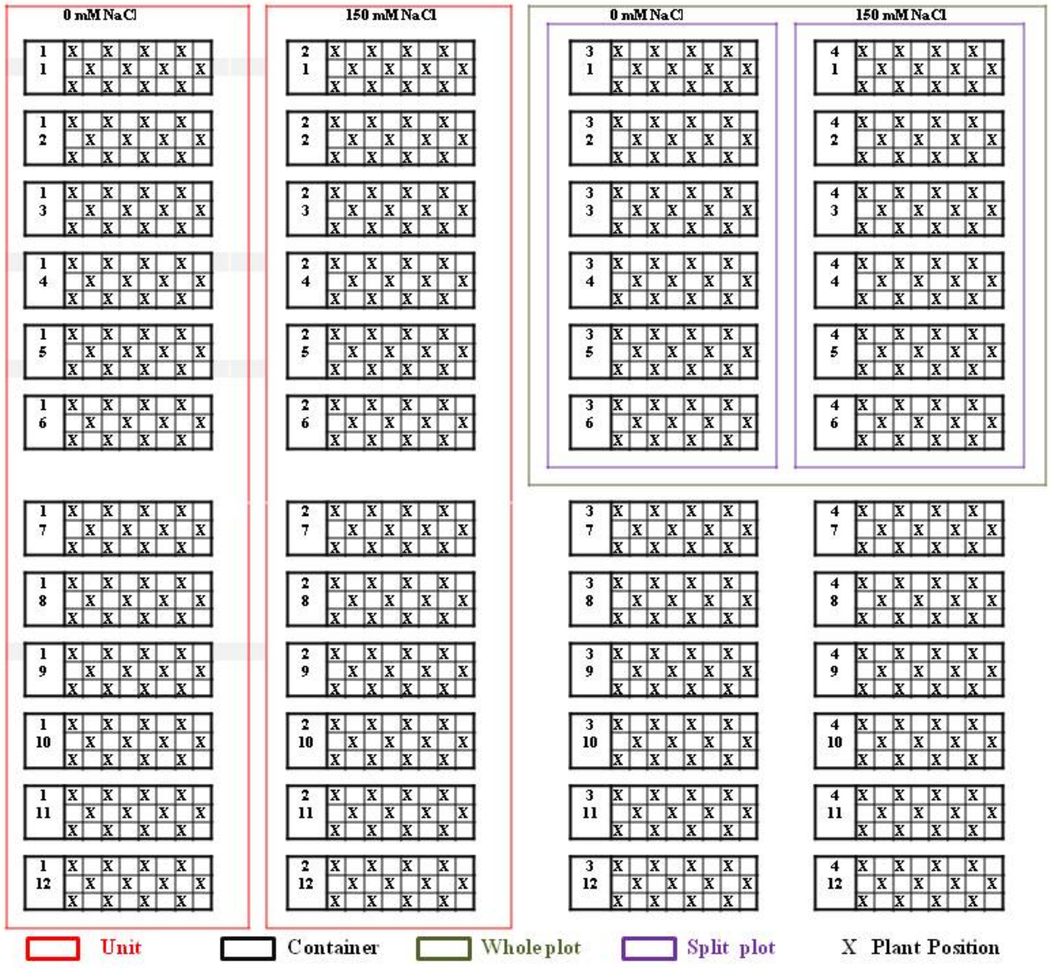

Supplementary Figure 1. Experimental design with 4 units for control and salt treatment. Each unit contained 12 containers for 2 replications with randomly distributed *Miscanthus* genotypes. The two treatments were assigned to one of the two units in a split plot. The whole plot contained 2 split plots and each split plot contained 6 adjacent containers.
