# Supplementary material for: Genetic Diversity of Salt Tolerance in Miscanthus
Source: Front Plant Sci. 2017 Feb 14;8:187. doi: 10.3389/fpls.2017.00187 (PMC5306379; doi:10.3389/fpls.2017.00187)
Supplement: Supplementary file 6 [file Image2.PDF]

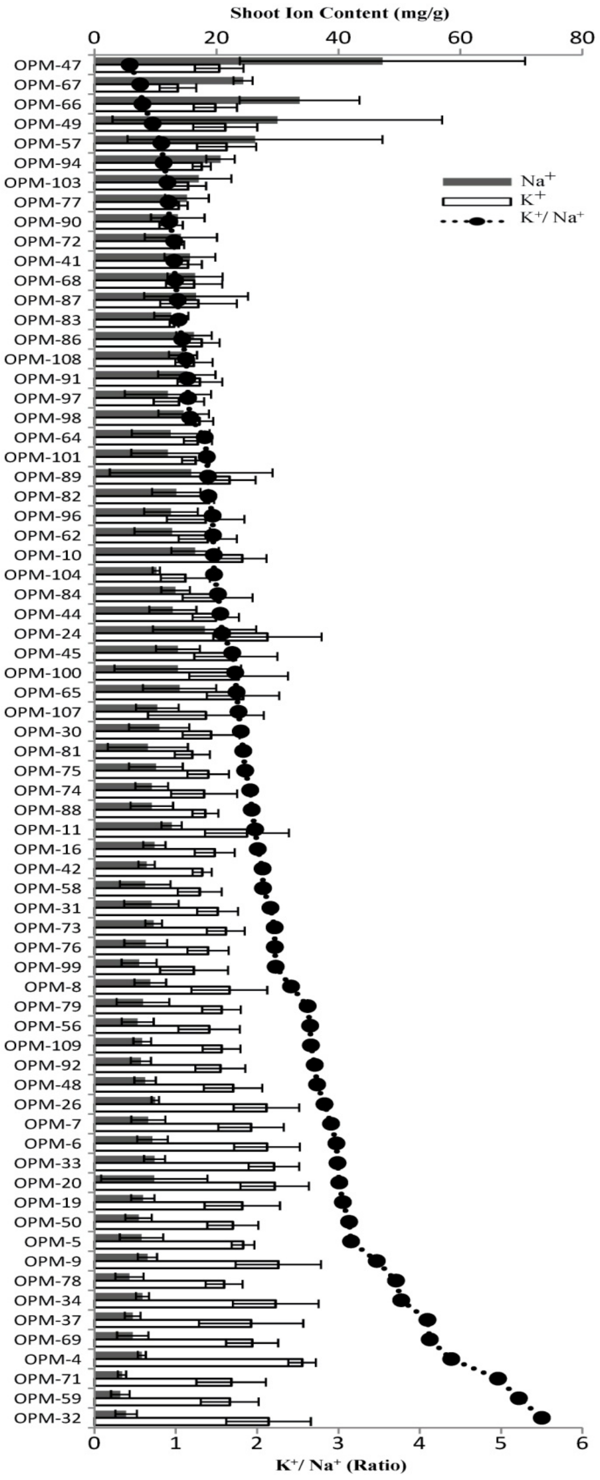

Supplementary Figure 2. Shoot Na^+ and K^+ concentration (gray and white bars, respectively) and shoot K^+/Na^+ ratio (line-scatter plot) in leaves of 70 *Miscanthus* genotypes grown under saline conditions (150mM NaCl). Error bars indicate standard deviation (n=4)
