# Supplementary material for: Genetic Diversity of Salt Tolerance in Miscanthus
Source: Front Plant Sci. 2017 Feb 14;8:187. doi: 10.3389/fpls.2017.00187 (PMC5306379; doi:10.3389/fpls.2017.00187)
Supplement: Supplementary file 7 [file Image3.PDF]

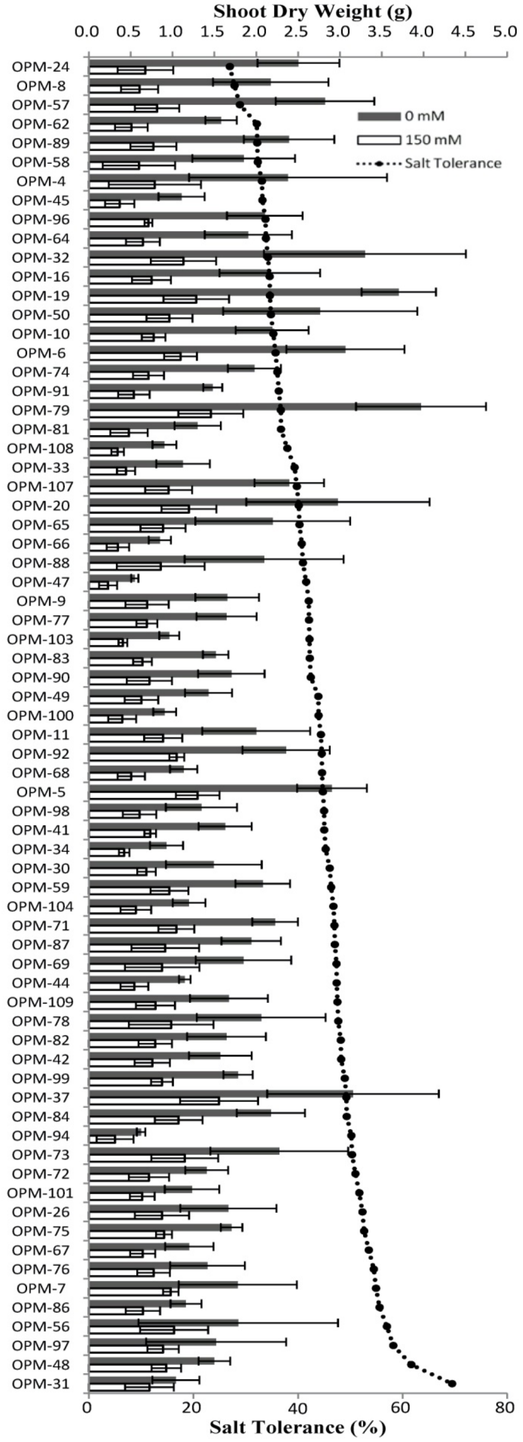

Supplementary Figure 3. Shoot dry weight (bars) and salt tolerance (line-scatter plot, calculated as the ratio of shoot dry weight under salt stress and shoot dry weight under control conditions) of 70 genotypes of *Miscanthus* grown in a hydroponics system at 0 mM NaCl (gray bars) and 150 mM NaCl (white bars). Error bars indicate standard deviation (n= 4).
